# A New Tool for Real-Time Pain Assessment in Experimental and Clinical Environments

**DOI:** 10.1371/journal.pone.0051014

**Published:** 2012-11-30

**Authors:** Nils Schaffner, Gerd Folkers, Silvia Käppeli, Markus Musholt, Günther F. L. Hofbauer, Victor Candia

**Affiliations:** 1 Collegium Helveticum, University of Zurich and ETH Zurich, Zurich, Switzerland; 2 Center for Nursing Research and Development, University Hospital Zurich, Zurich, Switzerland; 3 Department of Dermatology, University Hospital Zurich, Zurich, Switzerland; City of Hope, United States of America

## Abstract

Pain measurement largely depends on the ability to rate personal subjective pain. Nevertheless, pain scales can be difficult to use during medical procedures. We hypothesized that pain can be expressed intuitively and in real-time by squeezing a pressure sensitive device. We developed such a device called “Painmouse®” and tested it on healthy volunteers and patients in two separate studies: Sixteen male participants rated different painful heat stimuli via Painmouse® and a Visual Analog Scale (VAS). Retest was done one week later. Participants clearly distinguished four distinct pain levels using both methods. Values from the first and second sessions were comparable. Thereafter, we tested the Painmouse® by asking twelve female and male leg- ulcer patients to continuously squeeze it during the whole length of their wound-dressing change. Patients rated each step of dressing change on an 11-point numeric rating scale. Painmouse® ratings were highest for the wound cleaning and debridement step. Application of the new dressing was not evaluated as very painful. On the other hand, numeric scale ratings did not differentiate between dressing change steps. We conclude that the Painmouse® enables pain assessment even under difficult clinical circumstances, such as during a medical treatment in elderly patients.

## Introduction

Methods to accurately characterize patients’ pain are needed. Despite modern techniques, pain remains a subjective experience and health care professionals have to rely on patients’ ratings [1]. The VAS is simple, depends little on language and is commonly used [2]. Problems affecting scales like the VAS are position bias in the continuum [3], completion during or immediately after a medical procedure and increased failure rates in geriatric patients [4], [5], [6]. Armstrong et al. did seminal work to automatically record pain experiences with mechanical devices [7]. Nevertheless, experience was needed to discriminate different pain levels and discrimination was best only for moderate pain. As an alternative, we developed the Painmouse® (PM) [8], a human-computer interaction tool. Electronic pain assessment tools offer real-time evaluation, reducing missing data and ambiguous markings [9]. Concurrently, patients accept or even prefer such tools [10]. The PM easily fits into the hand measuring forces exerted upon it. The device captures the intuitive clenching reaction to pain, without visual feedback, and it can be used one-handed. Patients unable to move or with limited view can also use the PM.

Contrary to VAS, successive PM-ratings do not entail the risk of visual memories confounds. Therefore, in the first study we assessed the acceptance and validity of the PM compared to the VAS.

Dressing changes are known to be the most painful part in the everyday life of patients suffering from chronic wounds [11]. Patients who are well-informed about the treatment and who can communicate their pain express reduced stress and pain levels [12]. Older patients want care providers to take them seriously when they express pain [13]. Strikingly, few records document the pain of patients suffering from venous leg ulcers, and only few methods have been standardized [14], [15], in spite of international guidelines recommending pain assessment for all patients suffering from leg ulcers [16]. Moreover, healthcare practitioners often underutilize methods to control pain during treatment procedures [11], while almost a quarter of patients with chronic wounds felt their pain medication is ineffective [17]. Therefore, it is likely that an easy-to-use and reliable pain assessment tool would help practitioners to properly acknowledge the patients’ need for stronger pain medication or for wound dressing procedures designed to minimize pain. Furthermore, such a tool could improve the early detection of wound infections, since increased pain levels are a good indicator [18]. Furthermore, we hypothesized that leg ulcer patients can accurately indicate perceived pain intensity over the whole procedure of a wound dressing change via handgrip force.

### Methods Study 1

This study was conducted according to the guidelines of the Declaration of Helsinki for the treatment of experimental subjects. All volunteers gave written informed consent to the project, which was approved by the local Ethics Committee.

### Painmouse® (For Handgrip Measurements in Study 1 and 2)

**Figure 1:**
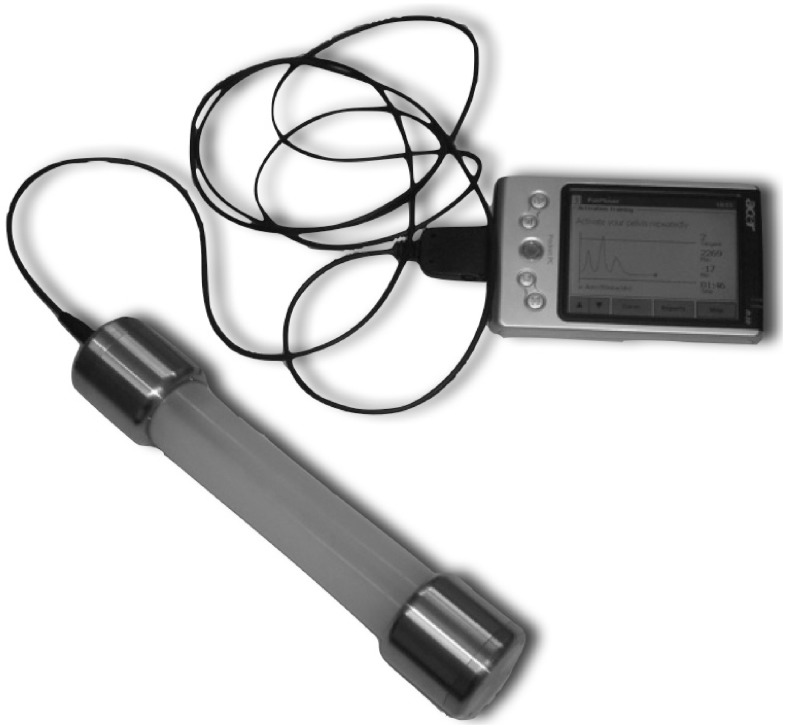
The Painmouse®. The measuring device connected to a hand-held portable computer.

The Painmouse® (PM) has a dumb-bell shape with a 13 cm long, and 3 cm in diameter silicon tube filled with silicon oil and equipped with a steel bolt in the middle. The bolt connects two stainless steel ends, each 3 cm in length with 4 cm in diameter. One of the ends contains a pressure sensor, which is wired to a hand-held portable computer (see Figure 1). During measurements, certain points in time can be marked manually for off-line analysis. Sampling rate for the pressure data was 10 Hz (pressure’s accuracy±0.1 mbar).

### Subjects

Sixteen healthy, right-handed male volunteers free of pain and any sensory abnormalities as stated in self-report, were recruited for the study. Handedness was determined using a standard handedness inventory [19]. Mean age of participants was 24.9 years (SD 7.01).

### Pain Stimuli

Noxious heat stimuli were administered to the left volar forearm using a 30×30-mm Peltier device (Medoc, Ramat-Yishai, Israel; TSA-II) placed at 2/3 of the distance from wrist to elbow. Individual pain threshold was measured using the method of limits: Participants were asked to press a button held in their right hand when the heat stimulus from the thermode, starting at 35°C and increasing with 1°C/sec, changed from just hot to painfully hot. The average of three such measurements was defined as the individual pain threshold (interstimulus interval = 20 sec). This pain threshold was used to determine the following individual heat stimuli: NO PAIN (threshold –1°C), LOW PAIN (threshold +1°C), MEDIUM PAIN (threshold +2°C), HIGH PAIN (threshold +3°C). Each of these 4 individual stimuli was randomly applied three times for the length of five seconds (interstimulus interval = 20 sec). To avoid physical injuries, subjects with an individual pain threshold higher than 47°C were excluded from the study. Prior to measurements, subjects were allowed to familiarize with the heat stimuli and the handling of the controlling device [20]. Of the 15 pain stimuli used in each run, the first three were of no, low and medium pain stimulus intensity. They were presented in randomized order to allow familiarization. Ratings pertaining to these three stimulus intensities were not used in any of the statistical analyses. PM data was screened for rating peaks with the aid of the markers set during the experiment at the beginning of each stimulus presentation. The highest value of each corresponding positive PM-slope deflection was used for further analyses.

### Experimental Procedure

Participants sat upright at a desk, reading the instructions via a computer monitor placed in front of them. Each experimental session comprised two runs consisting of a series of 15 individual pain stimuli, whose intensity was rated using a VAS in one run and the PM in the other. In order to minimize the influence of earlier VAS-ratings, each scale was presented on a separate paper sheet. VAS ends were marked with 0 indicating “no pain” and 10 “worst pain experienced”. Between the two runs there was a 15-minute break. To check for reproducibility, a second and identical session was carried out exactly one week later. Rating methods were randomized and counterbalanced for run and session. Subsequent to both runs participants rated how accurate, intuitive and complex the pain rating methods were using 10-cm VAS with end values of 0 indicating “not at all” and 10 as “very high”.

### Statistical Analyses

To evaluate the effect of pain levels and session (e.g., first week vs. second week) on VAS and PM-ratings, a two-factorial repeated measurement analysis of variance (ANOVA) with the factors *Pain* (no, low, medium and high) and *Session* (1 vs. 2) was computed. Prior to this analysis, the mean of the three stimulus presentations for any of the four pain level ratings was calculated.

Three two-factorial repeated measurements’ ANOVAs were computed on the questionnaire ratings on how accurate, intuitive and complex the two methods were perceived. These ANOVAs included the factors *Method* (VAS and PM) and *Session* (1 vs. 2). P-values in the ANOVAs were Greenhouse-Geisser corrected. Post-hoc comparisons were done using *t*-tests for dependent samples. P-values of pair-wise comparisons were adjusted using the false discovery rate method [21]. Significance level was set at P<0.05 for all statistical calculations other than post-hoc comparisons.

### Methods Study 2

This study was conducted according to the guidelines of the Declaration of Helsinki for the treatment of experimental subjects. All volunteers gave written informed consent to the project, which was approved by the local Ethics Committee.

### Subjects

Thirteen patients were enrolled for the study after giving informed consent orally and in writing. One patient only completed one clinical session and was not considered for further analyses. None of the patients suffered from dementia or chronic pain. (Supporting information Table S1). All patients understood they had to score the acute dressing-change-related pain and not baseline pain.

### Dressing Change

The dressing change was divided into the following five steps: 1) Removal of the old dressing 2a) Wound cleaning and/or disinfection, or 2b) debridement following pretreatment with local anesthetics (EMLA® cream), 3) Application of the new dressing, 4) Application of the compression dressing or compression stockings. Patients needing wound debridement did not receive a wound cleaning and vice versa. For this reason, all patients underwent only four steps during one ‘run’.

### Experimental Procedure

The patients sat with their back half upright on a bed with their legs stretched out. To ensure that not much muscular effort was needed to hold the weight of the device during the whole dressing change procedure, participants were asked to hold the PM using the hand that suited them best. The only instruction they received for the use of the device was to squeeze the PM according to the intensity of their pain sensation, for example to squeeze it strongly when they felt strong pain. The nurse then started the dressing change. After each of the predefined five steps mentioned above (see under dressing change), patients were asked additionally to rate the pain intensity felt during the preceding step using a number between 0 and 10, 0 meaning “no pain” and 10 meaning “worst pain” (Numerical Rating Scale, (NRS)). The NRS was used because it does not require any manipulations with the hands, which could distract the patients from using the Painmouse®. In order to check within-subject repeatability, the same patient rated his/her pain in three subsequent runs of dressing changes. The minimum time interval between two runs was 24 hours.

### Statistical Analyses

The PM data of each run was divided into the five different dressing change steps using the time markers set during the procedure. The highest value of each section was then used for further analyses. To check for possible differences due to gender and medication affecting the average pressure on the PM, PM values were standardized for each session by computing the average pressure of all the three parts rated by all patients (old dressing removal, application of the new dressing and application of the compression dressing). This value was then used to calculate the percentage of all the ratings of this session in relation to this value. To evaluate the effect of the different wound dressing steps on PM values, repeated measurements analyses of variance (ANOVA) with the within-subject factors *Step* (removal, wound treatment, new dressing, compression) and *Run* (first, second, third) and the between-subject factor *Gender* were calculated. Post-hoc comparisons were made by means of paired single *t*-tests. P-values of pair-wise comparisons were adjusted using the false discovery rate method [21].

Since patients were able to give only 81 out of 141 possible NRS ratings (see under discussion), the obtained values of all three runs for each patient were averaged prior to all calculations. Thereafter, an ANOVA comprising the within-subject factor *Step* (removal, wound treatment, new dressing, and compression) and the between-subject factor *Gender* was computed. Wound cleaning and debridement steps were merged to a “wound treatment” step after the independent samples *t*-test did not show any significant differences of NRS ratings between these two treatments.

### Results Study 1

Thirteen men (average age: 23.4 years (SD 4.88)) were included in all data analyses while three participants were excluded, apart from the analysis of their questionnaires. One of the excluded volunteers missed the second measuring session, one did not feel the stimuli as painful and the third misunderstood the given instructions.

### VAS

**Figure 2:**
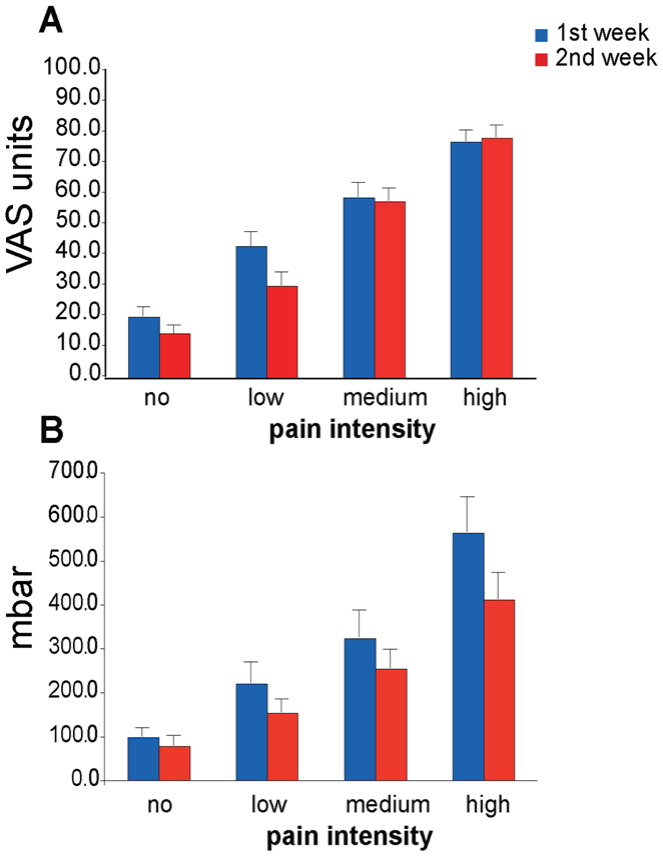
Medium pain ratings of four distinct pain levels (A) VAS (B) PM. All comparisons between the pain intensities were significant (p ≤ 0.05) for VAS and PM. Bars depict average values and their S.E. Please note that for the VAS, ratings for medium- and high-pain levels do not change over time in contrast to PM values for the same pain levels (see Discussion).

The ANOVA for pain levels as measured with VAS-ratings was significant for the within-subjects factor *Pain* (F(3,36) = 132.8, p≤0.000). Post-hoc *t*-tests showed that the low-pain level was higher than the no-pain level (t = −5.857, p≤0.000), the medium-pain level higher than the low-pain level (t = −9.012, p≤0.000), and the high-pain level higher than the medium-pain level (t = −8.667, p≤0.000). The factor *Session* was indifferent (F(1,12) = 1.705, p = 0.216), in contrast to the interaction Pain*Session (F(3,36) = 4.089, p = 0.027). Nevertheless, none of the relevant comparisons (e.g. high-pain level session 1 vs. high-pain level session 2) survived FDR-corrections. VAS-ratings in the low-pain levels revealed a tendency to decrease from the first to the second session (t = 2.303, p = 0.040 uncorrected). The averaged no-pain, and low-pain VAS-ratings decreased around 30% from the first to the second session (no-pain = −27.5%, low-pain = −29.8%) while the medium-pain and high-pain ratings remained stable (medium-pain = −2.1%, high = +1.7%) (See Figure 2A).

### PM

The ANOVA for pain levels as assessed with PM-ratings revealed differences (F(3,36) = 36.208, p≤0.000). Post-hoc *t*-tests showed that the low-pain level was higher than the no-pain level (t = −3.958, p = 0.002), the medium-pain level higher than the low-pain level (t = −3.946, p = 0.002) and the high-pain level higher than the medium-pain level (t = −7.088, p≤0.000). In contrast to the VAS-ratings, the factor *Session* showed differences between sessions (F(1,12) = 7.628, p = 0.017). PM-ratings in the second session were lower than in the first session. Also the interaction Pain*Session indicated differences (F(3,36) = 7.276, p = 0.004), but post-hoc *t*-tests revealed that none of the relevant comparisons (see this section under VAS) survived FDR-corrections. All averaged PM-ratings decreased constantly around 20–30% from the first to the second session (no = −21%, low = −30.4%, medium = −21.5%, high = −26.9%) (See Figure 2B).

### Questionnaire

**Figure 3:**
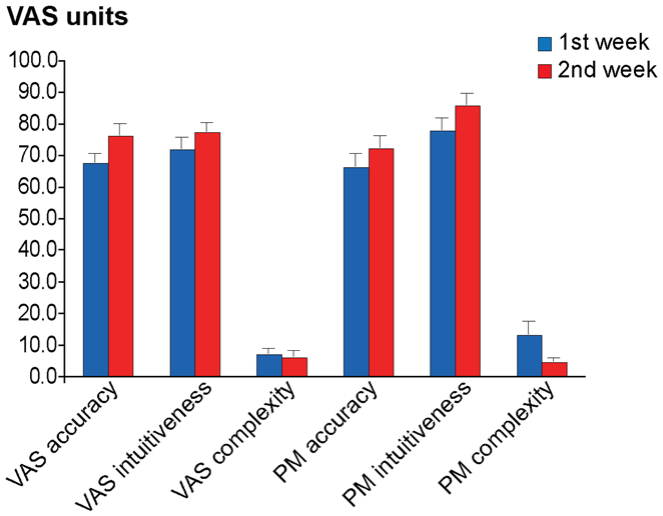
Mean complexity, accuracy and intuitiveness ratings of the VAS and the PM. There was no significant difference between the two methods or between ratings of the first and second week. However, there was a trend to rate the PM-method as being more intuitive. Bars depict average values and their S.E.

None of the ANOVAs for complexity (F(1,14) = 0.358, p = 0.559), accuracy (F(1,14) = 0.071, p = 0.794) and intuitiveness ratings were indicative of relevant differences, yet there was a statistical trend in the within-subjects factor *Method* of the intuitiveness rating (F(1,14) = 4.230, p = 0.059), whereby participants show a trend to rate the PM-method as being more intuitive than the VAS-method (See Figure 3).

### Results Study 2

Twelve patients, six women and six men visiting the Dermatological Clinic of the University Hospital Zurich and suffering from painful leg ulcers participated in this study. The mean age of the participants was 70.5 years (SD 16.34). Six women (average age = 73.0 years SD = 19.83) and six men (average age = 68.0 years SD = 13.38) were included in all data analyses. While during 22 dressing changes the wound was only cleaned and disinfected, debridement was carried out 14 times. The independent samples *t*-test showed no significant differences of PM values between these two sorts of treatment (t = −0.859, p = 0.407). For the calculation of an ANOVA over all patients and runs, these values were merged to a “wound treatment” step. Three values pertaining to the removal step were missing because three patients had already removed the old dressing prior to the official dressing change. Missing values were from three different runs. To fill in the data set, the arithmetic mean of the two remaining values of each patient was used.

### PM

**Figure 4:**
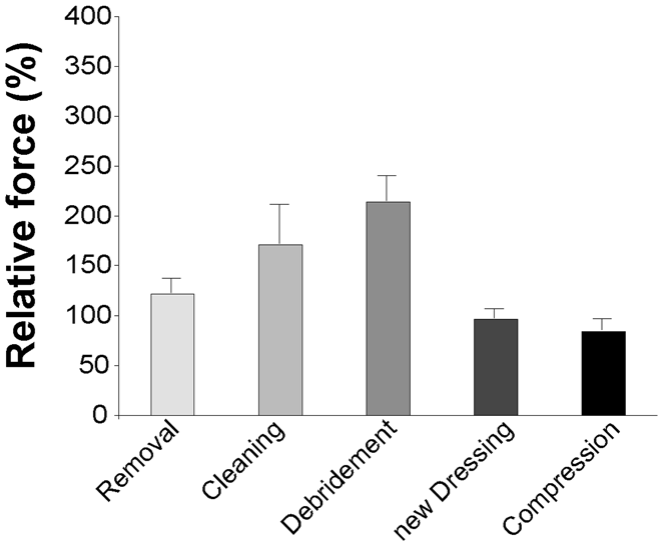
Patients’ standardized mean PM ratings of the five different dressing change steps. Bars depict average values and their S.E.

The ANOVA for pain values via PM was significant for the within-subjects factor *Step* (F(3,30) = 6.812, p = 0.007). Post-hoc *t*-tests showed that PM values for the wound treatment step were higher than those during the removal step (t = −3.322, p = 0.021), the new dressing step (t = 2.772, p = 0.036) and the compression step (t = 3.416, p = 0.036) (see Figure 4). The factor *Run* (F(2,20) = 1.951, p = 0.170) as well as the between-subject factor *Gender* (F(1,10) = 1.287, p = 0.283) or any interaction were indifferent, meaning that pain values of the three consequent dressing changes in each patient were stable and female and male patients rated the pain during dressing change as equally painful. To ensure that the latter two results were not a consequence of data standardization, an ANOVA, including the original values, was calculated. Identical to the ANOVA for standardized values, this new ANOVA was significant for the within-subjects factor *Step* (F(3,30) = 4.354, p = 0.026) but not so for the factor *Time* (F(2,20) = 0.376, p = 0.683) or any interaction. The between-subject factor *Gender* (F(1,10) = 4.016, p = 0.073) showed a trend of female patients to have higher PM values.

### NRS

The ANOVA for pain ratings using the NRS did not reveal any differences, neither for the within-subjects factor *Step* (F(3,21) = 2.422, p = 0.147) nor for the between-subject factor *Gender* (F(1,7) = 1.342, p = 0.285).

## Discussion

We compared a new pain measurement device with a commonly used VAS. Both methods accurately discriminated four distinct heat-pain levels administered to the forearm of healthy participants. Participants were unaware of the number of levels and intensity of pain stimuli, but they used the whole range of the VAS for their ratings. Apparently, our protocol induced pain from low to high levels, encompassing the full range of heat-pain stimuli. The PM-ratings of all pain levels decreased by 20 to 30% from first to second session, in agreement with other studies [22], [23]. The decrease was most probably due to experimental adaptation. Interestingly, the VAS-ratings of the no- and low-pain level decreased similarly to the PM-ratings, but not so the medium- and high-pain level ratings. It is possible that participants’ visual memories of the high ratings on the VAS-scale were at work here. To explore this issue, we studied this effect in a side experiment (Supporting information S): 16 participants rated the size of small, medium and large circles using a VAS and the PM (Supporting information Figure S1). After a week, they were asked to repeat their rating from memory. In agreement with our assumptions, only the VAS rating for the memories of the large circle did not differ significantly from the previous rating. The VAS rating for the small circle was significantly lower (Supporting information Figure S2A), while the recall of the PM ratings was significantly higher (Supporting information Figure S2B). Based on results of this side experiment, we conclude that it is likely that the decreased PM ratings during the main study were a direct translation of the pain the participants experienced in the second session, in contrast to the VAS for the medium- and high-pain level ratings. The sampling rate for the PM was 10 Hz. While low, this sampling rate permitted to record each pain stimulus rating, and to display it as a two-dimensional peak deflection along a curve trajectory. There are two reasonable ways of reading the ratings of each pain stimulus: a) to read the maximal pressure and b) to calculate the integral associated with a particular force output. While the first can easily be determined, the second might be a more accurate estimate, as the time span of the experienced pain is included. We saw an almost perfect correlation between the two possible readouts, which shows that both calculations are, in this case, equally informative (data not presented). Therefore, we suggest using the maximal pressure values for similar calculations.

A particularly interesting question is whether it would be possible to generally assign a certain handgrip force to a distinct pain level. The relatively small number and the selection of subjects in the first study can only give a limited answer to this question. Nevertheless, as each subject was measured twice and each pain level was administered 3 times, a total of 78 ratings per pain level were assessed. It is probable that for young men, the range of pain levels and their corresponding handgrip forces will range between 0 to 100 mbar for no-pain, and between 400 to 600 mbar for high-pain. This should be assessed in the future.

We hypothesized that the VAS-method would be experienced as more accurate than the PM-method because participants could see their pain estimate, allowing the reevaluation of their scores. On the other hand, it was hypothesized that the PM would be experienced as more intuitive, as there was no visual feedback and therefore no need to convert the experienced pain into a particular value on a given scale. Accuracy and intuitiveness were rated as being high in the two methods and sessions. However, there was a trend to higher rates for intuitiveness for the PM. The complexity of both methods was rated as being very low and similar within and between sessions. We believe that the real utility of the PM as an assessment method in experimental settings is the automaticity of the procedure, the avoidance of missing values, the avoidance of visual confounders, and the new possibility to explore objectively differences in force output ranges in relation to pain in different cohorts displaying diverse ranges of force output (e.g., children, young and elder people, men an women). The latter opens a potential window of opportunity to describe pain experiences with a finer resolution.

Leg ulceration is most painful at dressing changes [24]. For the majority of patients this pain is the worst part of living with a wound [17]. Still, only a few health care providers use standardized pain assessment tools [15]. More importantly, they often do not believe that their patients are in pain [25]. In addition, pain medication and pain reducing dressings are under-used [11]. Two major reasons may account for this: First, the nurse – often being under time pressure – has to focus on the dressing change. Second, and crucial for the present research, so far no pain assessment method that would be easy to use when working with elderly patients during a medical procedure has been available. The Painmouse® addresses both shortcomings. Using the PM, patients in this series expressed the most intense pain during the debridement and wound cleaning and/or disinfection part. The next painful intervention was the removal of the dressing. Exactly the same ranking pattern can be found in other recent research reports [17]. Conversely, applying the new dressing seemed to have caused nearly no pain. This is consistent with reports showing that covering a wound leads to pain reduction [26]. Furthermore, the compression part was rated to be not or only a little painful. Wound-pain specialists recommend using the NRS during dressing changes because it is supposedly easier to explain, particularly to elderly patients [27]. Nevertheless, a third of the NRS ratings were missing. Strikingly, patients declared they did not want to say something wrong. For example, for one patient, the 11-point NRS had too many choices to deal with. Thus, such problems were most probably the main reason why NRS ratings showed no significant differences between the dressing change steps.

Since the pain assessment method developed here is based on a motor response (squeezing a pressure sensitive device), there are potential limitations for its use in pain-patients with motor deficits (such as Parkinson's disease) but also in those patients showing a deficit in cutaneous sensory functions (e.g. in painful polyneuropathy), which may influence the squeezing response. While future work may shed light on these aspects, the amount of pain-patients not belonging to the named categories can be considered to be large. Consequently, there is a wide patients’ spectrum where the PM can be used.

Before starting into a large-size clinical study, we chose a strategy of smaller sequential studies for proof-of-concept and the possibility to improve later studies by experiences gained. Importantly, since the aim of pain detection is to treat it, the used measurement technique must be sensitive to changes in pain secondary to treatment. We assessed the influence of the strength of pain medication on PM values and NRS ratings. While our data is preliminary in nature, ordering the different pain killers taken 24 hours previous to the dressing change (Supporting information Table S1), from weak to strong, to study the influence of the strength of pain medication on PM values and NRS ratings indicated that mean PM values correlated inversely with the strength of painkillers taken during the 24 hours previous to the dressing change (Supporting information Figure S3). Conversely, this was not true for NRS ratings. We interpret this fact as a potential indication for the sensitivity of the PM to changes in pain treatment (Supporting information Figure S4).

In our opinion, the major limitation of the present studies is that PM-values are a relative rather than an absolute measure. While the maximum handgrip force in each patient was assessed, equalizing this value with the worst ever experienced pain is meaningless considering that the maximum handgrip force appears to be several times higher than the reported average pain rating. In the first study we found that a pressure of 150–200 mbar equals low-pain, corresponding to the average pressure recorded by the leg ulcer patients during painful treatment steps. In future research, patients should rate a painful stimulus before pain assessments. By using this stimulus as a reference, the PM could be calibrated accordingly; therefore ratings could be translated to a 101-point scale or/and a verbal rating scale.

### Conclusion

Although some of our data is preliminary, we show that the PM tool distinguishes different levels of pain. Patients expressed their pain intuitively via a force-recording device, without the risk of missing values. A particular advantage is that nurses using the PM do not need to attend to pain assessment concurrently to patient care. Beyond patients of the kind presented here, other patients such as children may also benefit from a better pain assessment during painful medical treatment. In addition, pain research may profit from the use of a device like the Painmouse® while quantifying pain.

## Supporting Information

Figure S1 **Stimulus material, side experiment.** Small, medium and large circles on a black, quadratic background.
(TIF)

Figure S2 Average size ratings (small and large circle) and corresponding force memory of the given ratings after one week. A) VAS ratings B) PM ratings. Bars depict average values and their corresponding standard errors.
(TIF)

Figure S3 **Pain medication.** Mean PM values correlated negatively and significantly with the strength of painkillers taken during the 24 hours previous to the dressing change (Spearman’s Roh = -0.868, p≤0.000).
(TIF)

Figure S4 **Pain medication.** Mean NRS values did not significantly correlate with the strength of painkillers taken during the 24 hours previous to the dressing change (Spearman’s Roh = −0.083, p<0.809).
(TIF)

Table S1 **Patients’ demographic information Study 2.**
(DOCX)

Text S1 **A side experiment.**
(DOCX)
