# Supplementary figures and images for: A New Tool for Real-Time Pain Assessment in Experimental and Clinical Environments
Source: PLoS One. 2012 Nov 30;7(11):e51014. doi: 10.1371/journal.pone.0051014 (PMC3511427; doi:10.1371/journal.pone.0051014)

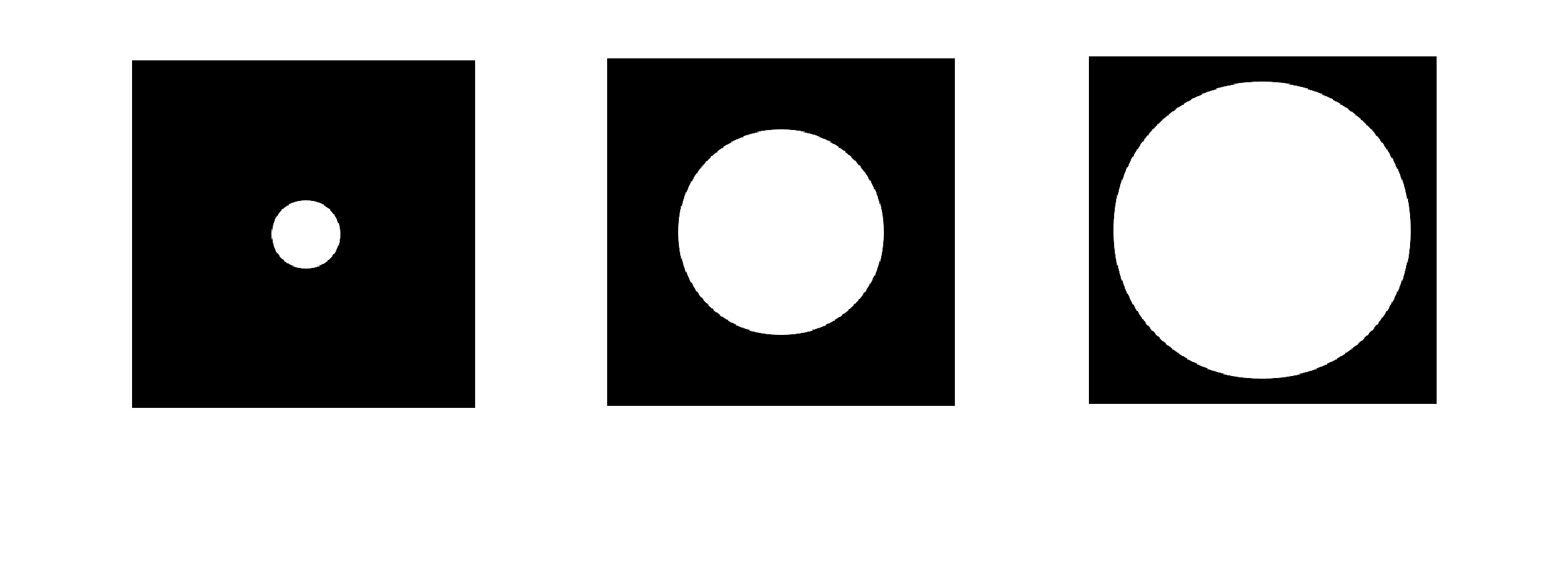

Supplement: Figure S1 — Stimulus material, side experiment. Small, medium and large circles on a black, quadratic background. (TIF) [file pone.0051014.s001.tif]

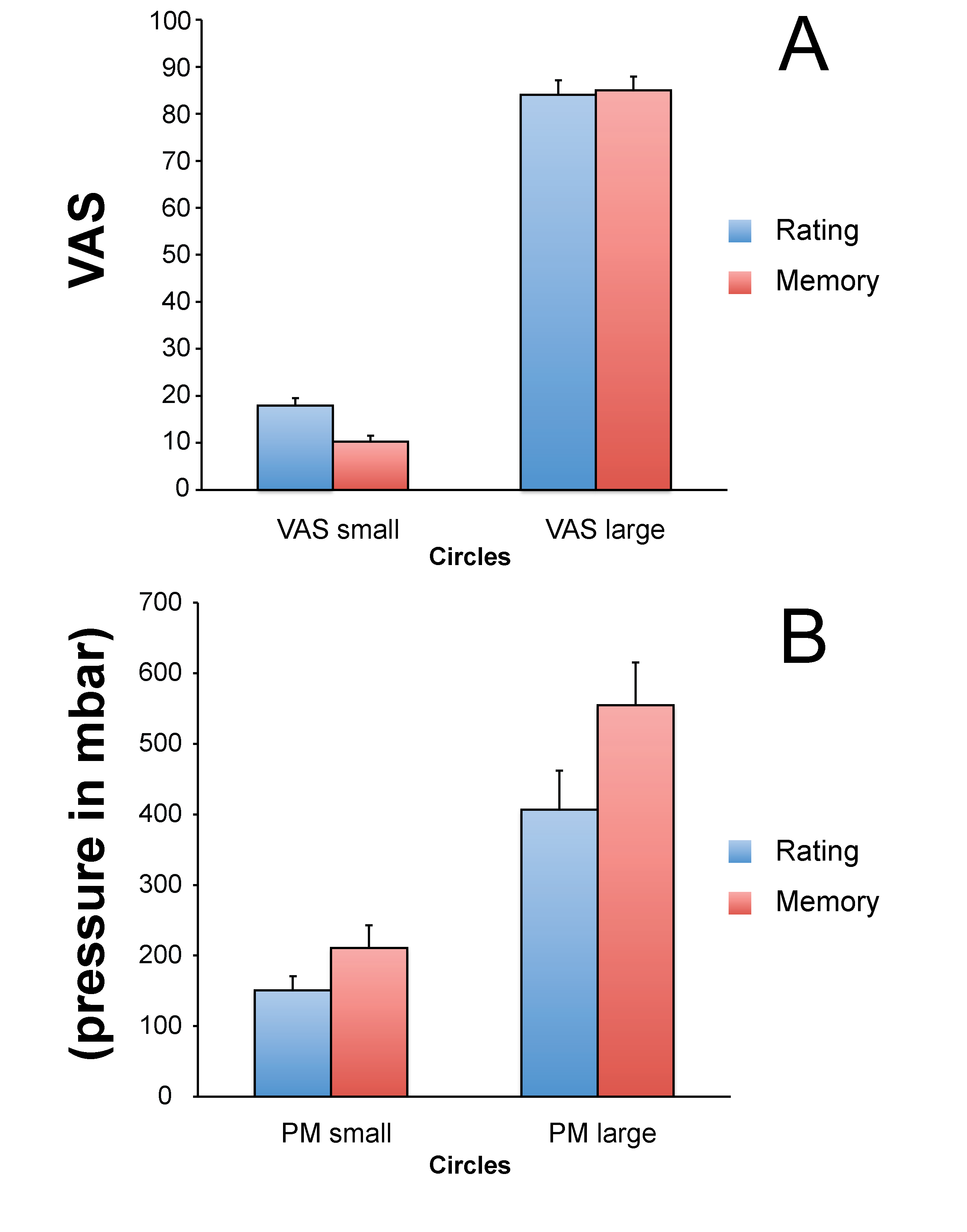

Supplement: Figure S2 — Average size ratings (small and large circle) and corresponding force memory of the given ratings after one week. A) VAS ratings B) PM ratings. Bars depict average values and their corresponding standard errors. (TIF) [file pone.0051014.s002.tif]
