# Supplementary material for: A New Tool for Real-Time Pain Assessment in Experimental and Clinical Environments
Source: PLoS One. 2012 Nov 30;7(11):e51014. doi: 10.1371/journal.pone.0051014 (PMC3511427; doi:10.1371/journal.pone.0051014)
Supplement: Table S1 — Patients’ demographic information Study 2. (DOCX) [file pone.0051014.s005.docx]

| **Patients** | **Gender** | **Age** | **Ulcus** | **Length of Ulcus in months** | **Reported Medication (all 3 sessions merged)** | **Assigned strength values of medication** | **Average strength of medication (all 3 sessions)** | **Average PM (all 3 sessions)** | **Average NRS (all 3 sessions)** |
| --- | --- | --- | --- | --- | --- | --- | --- | --- | --- |
| UCP_01 | Male | 72 | arterio-venous | 50 | Paracetamol, Metamizol, Tramadol, Hydromorphon | 1, 2, 3, 4, | 2.5 | 84.38 | 4 |
| UCP_02 | Female | 74 | venous | ca. 156 | - | 0 | 0 | 179.83 | 3 |
| UCP_03 | Female | 78 | arterio-venous | ca. 120 | Paracetamol | 1 | 1 | 152.58 | 3 |
| UCP_04 | Female | 78 | trauma | 1 | Paracetamol, Tramadol | 1, 3 | 2 | 139.64 | 6 |
| UCP_05 | Female | 86 | hypertonicum Martorell-Syndrome | 2 | - | 0 | 0 | 169.06 | 2 |
| UCP_06 | Male | 73 | arterial | 1 | Paracetamol, Tramadol | 1, 3 | 2 | 151.58 | 1 |
| UCP_07 | Male | 85 | trauma multifactorial | 6 | Paracetamol | 1 | 1 | 148.23 | 7 |
| UCP_08 | Female | 88 | arterio-venous | 14 | Paracetamol, Metamizol | 1, 2 | 1.5 | 143.17 | - |
| UCP_09 | Female | 34 | antibodies syndrome | 25 | Tramadol | 3 | 3 | 126.08 | 3 |
| UCP_10 | Male | 47 | venous | ca. 60 | Paracetamol, Metamizol | 1, 2 | 1.5 | 161.16 | 4 |
| UCP_11 | Male | 58 | hypertonicum Martorell-Syndrome | 13 | Paracetamol, Metamizol, Fentanyl | 1, 2, 4 | 2.33 | 28.26 | 2 |
| UCP_12 | Male | 73 | arterio-venous | 6 | Tramadol | 3 | 3 | 67.35 | 2 |

Table 1 S. Patients’ demographic information Study 2.
