# Supplementary material for: A New Tool for Real-Time Pain Assessment in Experimental and Clinical Environments
Source: PLoS One. 2012 Nov 30;7(11):e51014. doi: 10.1371/journal.pone.0051014 (PMC3511427; doi:10.1371/journal.pone.0051014)
Supplement: Text S1 — A side experiment. (DOCX) [file pone.0051014.s006.docx]

**Supporting information S**

**Introduction**

There is wide consensus that memories of a specific rating on a rating scale can influence the following ratings on the same type of scale. Therefore, physically different but conceptually related types of scales (e.g. first an 11-point numerical rating scale, then a 10-cm visual analog scale) have been used, when remembering the experience and not the previous rating was the goal ([Eich et al., 1985](#_ENREF_1))

In this side experiment we wanted to test to which extent remembering a rating given by handgrip force (using the Painmouse^®^) is possible. In addition, we compared the outcome with results obtained my means of the VAS, as we assumed that visual memories might confound some VAS ratings seen in Study 2.

**Methods**

Sixteen healthy volunteers were recruited for this study (average age = 36.5 years, SD = 13.0). Nine women (average age = 33.1 years, SD = 12.4) and seven men (average age = 40.9 years, SD = 13.3) were asked to rate the size of small, medium and large circles on a quadratic black background using a VAS and the PM (see Figure S1). Each size was presented three times in randomized order. The three circles were presented to create a sort of continuum, as is the case for the VAS. As we assumed visual confounds at the higher end of the VAS and possible force grip memories for the PM (see Figure 2 of main study), we tested only the two extreme circles after a time interval of one week. Participants were asked to recall the rating of the smallest and largest circle using again the VAS and PM. Participants were explicitly asked to recall their rating and not the sizes of the circles. To evaluate if there is a difference between the actual ratings of the circle sizes (average of the three repetitions per size) and the memory of these ratings, we used paired samples t-tests with a significance level of p<0.05.

- Insert Figure S1 at about here -

**Results**

VAS: T-tests revealed that the recalled values of the small circle’s rating were significantly lower than the actual rating a weak before (t = 5.137, p ≤ 0.000), while there was no significant difference between recall and the actual rating for the large circle (t = -0.220, p = 0.829) (see Figure S2A).

PM: T-tests showed that the recalled values were significantly higher for both, small (t = -3.171, p = 0.006) and large (t = -4.165, p = 0.001) circles than the actual rating one week before (see Figure S2B).

- Insert Figures S2A and S2B at about here -

**Discussion**

This side study showed that participants trying to reproduce a rating, which was given by squeezing the Painmouse^®^, are not able to recall the force output they used for this rating a week before. On average, they produced about 40% more force output while trying to recall their first rating from the previous week. As no clear recall of preceding PM ratings was observed, it is likely that subsequent PM ratings were direct measurements of the experienced sensation, free of memory confounds. In contrast, we could verify what has been discussed in literature ([Eich et al., 1985](#_ENREF_1)) that ratings on a visual analog scale can be memorized and therefore are likely to influence following ratings.

**Literature**

Eich E, Reeves JL, Jaeger B, Graff-Radford SB. Memory for pain: relation between past and present pain intensity. Pain 1985;23: 375-380.
